# Reproducibility of Oxford Scoring in IgA Nephropathy: Is the Noise Due to an Educational Gap?

**DOI:** 10.34067/KID.0000000000000234

**Published:** 2023-08-31

**Authors:** Leal C. Herlitz

**Affiliations:** Cleveland Clinic, Cleveland, Ohio

**Keywords:** GN, IgA nephropathy, kidney biopsy, pathology, renal biopsy, renal pathology

Since its publication in 2009, the Oxford classification has become the most widely used and accepted pathological classification of IgA nephropathy.^1,2^ Aims of the Oxford classification included identifying pathologic variables with acceptable interobserver reproducibility that also had value independent of clinical parameters to guide clinical management and provide prognostic information. The development of clear definitions that could be successfully applied in routine clinical practice was an area of focus. The original classification identified mesangial hypercellularity (M), endocapillary hypercellularity (E), segmental sclerosis (S), and interstitial fibrosis/tubular atrophy (T) as having independent value. A subsequent update from the Oxford group in 2016 recommended that crescent formation (C) be added to the MEST score.^3^

In this issue of *Kidney360*, Howie and Lalayiannis present a systematic review of the Oxford classification aimed at assessing its reproducibility and prognostic value.^4^ What distinguishes this review from previous reviews is the focus on reproducibility of the MEST-C scoring. The most commonly used assessment of reproducibility encountered was use of intraclass correlation coefficients, expressed by the kappa statistic. Kappa values range from 0 (no agreement) to 1 (perfect agreement). The Oxford group proposed that a kappa score <0.40 indicates poor interobserver reproducibility, 0.40–0.59 represented moderate agreement, 0.60–0.79 was substantial agreement, and >0.80 was outstanding.^2,3^ What becomes apparent in this systematic review is that kappas for MEST-C scoring vary widely across studies. Several single institution studies showed scores ranging from moderate to outstanding^5–7^ while multi-institutional studies showed lesser agreement.^8,9^

Among the single-center studies reviewed, one small study from Japan assessed agreement of the Oxford parameters in 45 biopsies that were reviewed by five nephrologists.^6^ Kappa scores were 0.409 for M, 0.489 for E, 0.533 for T and 0.556 for C (moderate agreement), and 0.616 for S (substantial agreement). A single-center study from China reviewing 410 patients by three pathologists who were blinded to clinical data revealed kappa scores ranging from 0.63 to 0.68 for M, 0.56–0.67 for E, 0.6–0.75 for S, and 0.74–0.75 for T, indicating moderate to substantial agreement for each parameter examined. The highest kappa scores reported come from a Chinese study aiming to compare the reproducibility and prognostic values of the Oxford classification, Haas classification, and Lee score. Scores from two experienced pathologists in 412 biopsies showed kappa scores of 0.77 for M, 0.74 for E, 0.86 for S, 0.86 for T, and 0.84 for C, correlating with substantial to outstanding agreement for each Oxford component.

In contrast to the single-center studies, a large multicenter study from Japan which examined 411 biopsies from 50 facilities, examined by five different pathologists, showed moderate agreement for M (0.45) and T (0.45) but poor agreement for E (0.37) and S (0.39).^9^ The Oxford group published their experience comparing over 1100 MEST-C scores by local pathologist from 55 centers with that of a central pathologist in the VALidation of IGA cohort and examined the potential effect of variability on the prognostic value of the classification.^8^ This study captured, in detail, the variation between local review in central view. Agreement assessed by kappa was moderate for S (0.51) and T (0.53) and poor for M (0.28), E (0.19), and C (0.24). Specifically, they found more liberal diagnosis of M, E, and C lesions by local pathologists and less frequent diagnosis of S lesions compared with central review. It is worth pointing out that the central pathologists in the study were blinded to clinical data and to the local pathologist read. Conversely, local pathologists typically had access to clinical data, which may have introduced some bias. Biopsies scored by the local pathologist as E1, but by the central viewer as E0, occurred in patients with more proteinuria and lower GFR. Biopsies that were scored as E1 or C1/2 by the local pathologist were associated with subsequent use of immunosuppression. Also worth noting is that central reviewers did not have access to all the slides that were used by local pathologists, only to a single periodic acid–Schiff–stained slide in almost all cases. For focal lesions, this could have resulted in underdiagnosis by central reviewers.

The lack of concordance in the Oxford scoring in large multi-institutional studies is of significant concern because without reproducibility, true validation of the importance of lesions included in the classification and the effect of interventions in clinical trials become difficult to assess. While it is tempting to dismiss the single institution studies as ungeneralizable and overly optimistic, the gap in agreement between multi-institutional and single-center studies suggests that the underlying issue may essentially be one of training and iterative collaboration. While the definitions in the Oxford classification sound simple, their application in the real world is less straightforward. Working through difficult cases with colleagues to obtain consensus, or training through educational modules, may significantly improve performance of the classification across institutions.

There is precedent for this education-driven approach to increase agreement in other areas of pathologic scoring. Reproducibility of Gleason grading, widely used in evaluation of prostate biopsies and relied on for clinical decision making and as a prognostic tool in prostate cancer, has been assessed in many studies. Gleason scoring by specialized urologic pathologists may differ from scoring rendered by general pathologists, resulting in the selection of inappropriate therapy. One study enrolled 755 patients over a period of 2 years undergoing prostate needle biopsies that were read first by general pathologists and then by two urologic pathologists who were blinded to the original interpretations.^10^ Concordance rates of Gleason scores between the general and specialist reads improved progressively from 47.5% in the first 6-month period (kappa of 0.55) to 78.7% in the final 6 months of the study (kappa of 0.84). The general pathologists were provided with the reports generated by the urologic pathologists and used that feedback to better align their interpretations for future cases. Many other studies using educational tools such as lectures and digital microscopy sets to improve performance in Gleason grading were also reviewed in this study.^10^ This experience with Gleason grading suggests that similar efforts aimed at increasing concordance among renal pathologists in evaluating Oxford variables may be possible through increased education and collaboration. The rapidly expanding use of digital pathology and artificial intelligence to aid pathologists in performing tissue analysis through unbiased cell detection and classification may play an important role in achieving these higher levels of concordance moving forward. Improving the reproducibility of scoring across the parameters identified in the Oxford classification is essential to providing a valuable tool for guiding therapeutic decisions, assessing outcomes of clinical trials and predicting renal outcomes.
